# Toward Mobile Neuroimaging: Design of a Multi-Modal EEG/fNIRS Instrument for Real-Time Use

**DOI:** 10.3390/s26041342

**Published:** 2026-02-19

**Authors:** Matthew Barras, Liam Booth, Anthony D. Bateson, Aziz U. R. Asghar, Mehdi Zeinali, Adeel Mehmood

**Affiliations:** 1 School of Engineering and Technology, Faculty of Science and Engineering, University of Hull, Hull HU6 7RX, UK; m.r.barras-2014@hull.ac.uk (M.B.); liam.booth-2014@hull.ac.uk (L.B.); m.zeinali@hull.ac.uk (M.Z.); 2 Independent Researcher, Hull HU6 7RX, UK; tonybateson777@gmail.com; 3 Department of Psychology, University of York, York YO10 5DD, UK; aa516@york.ac.uk; 4 School of Digital and Physical Sciences, Faculty of Science and Engineering, University of Hull, Hull HU6 7RX, UK

**Keywords:** EEG, fNIRS, STM32H7, ESP32-S2, ADS1299, TLC5940, wireless brain monitoring, embedded system, mobile neuroimaging, Lab Streaming Layer

## Abstract

In this study, we present the design and development of a mobile, multi-modal electroencephalography and functional near-infrared spectroscopy (EEG/fNIRS) device for wireless neurophysiological monitoring. The system was engineered to achieve high signal fidelity, low power consumption, and a fully untethered operation suitable for ambulatory brain research. The device integrates four Texas Instruments ADS1299 24-bit biopotential amplifiers, providing up to 32 simultaneous acquisition channels. Signal control, processing, and local storage via an SD card are managed by an STM32H7 microcontroller, while an ESP32-S2 module handles Wi-Fi communication. Dual-wavelength light-emitting diodes and OPT101 photodiodes form the optical front-end, driven by digitally controlled constant-current sources for stable illumination. The design employs galvanic isolation, multi-rail power management, and a four-layer PCB layout to minimise interference between analogue, power, and digital domains. Data are captured by a deterministic, clock-driven STM32 acquisition loop and forwarded to the ESP32, which operates under an RTOS and streams packets over Wi-Fi for collection on a mobile phone or PC using the Lab Streaming Layer (LSL) framework. The STM32H7 architecture was chosen for its capability to support future embedded edge-machine-learning functions, enabling on-device signal quality assessment and artefact rejection. Validation demonstrations include 32-channel synchronised acquisition using the ADS1299 internal test signal, eyes-open/eyes-closed alpha modulation visualised in EEGLAB, a forehead fNIRS breath-hold response with physiological spectral content, and real-time ECG/optical pulse streaming via LSL. The resulting system provides a compact platform with explicitly defined acquisition and data interfaces for synchronised EEG/fNIRS acquisition, enabling scalable, low-cost mobile neuroimaging research.

## 1. Introduction

Mobile neuroimaging enables the study of brain dynamics in naturalistic and ambulatory settings, where traditional tethered instrumentation is impractical. Electroencephalography (EEG) provides millisecond-scale access to electrophysiological activity, while functional near-infrared spectroscopy (fNIRS) captures slower haemodynamic responses [1]. Simultaneous EEG and fNIRS acquisition enables investigation of neurovascular coupling and complementary neural dynamics, but imposes stringent requirements on timing, noise performance, and system synchronisation. Hybrid EEG–fNIRS systems have therefore emerged as a promising approach, combining the high temporal resolution of EEG with the spatial specificity and robustness of fNIRS to improve neural decoding performance in mobile and real-world applications [2].

Many existing EEG and fNIRS systems rely on proprietary hardware, loosely coupled acquisition pipelines, or independent timing domains for electrophysiological and optical signals. These design choices complicate hardware-level synchronisation, limit access to raw data, and restrict modification of acquisition parameters [3,4,5]. For mobile systems, wireless transport and power management further exacerbate timing jitter and noise coupling if acquisition and communication are not carefully partitioned.

This work builds upon the smartphone-based EEG platform previously developed by Bateson and Asghar [6], extending the architecture to a battery-powered EEG/fNIRS instrument that prioritises hardware-timed acquisition, synchronised multimodal sampling, and explicitly defined acquisition and data interfaces compatible with established neurophysiology software workflows.

**Figure 1:**
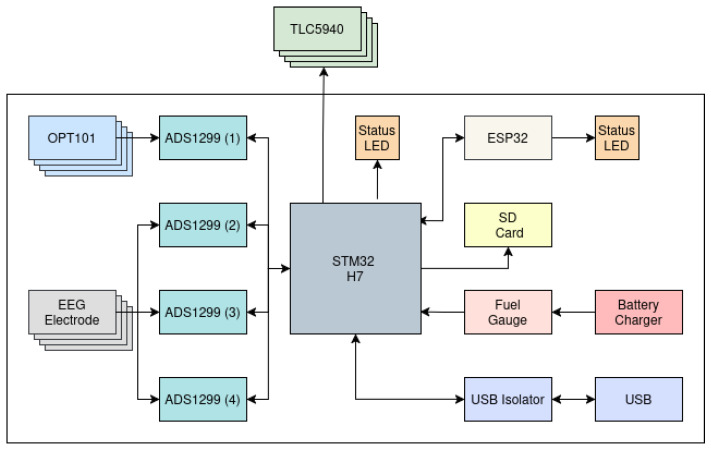
Block-level diagram of the complete signal, timing, and power architecture.

Figure 1 illustrates a block-level diagram of the complete signal flow. EEG electrodes and optical detector outputs (OPT101) are digitised using four synchronised ADS1299 biopotential ADCs sharing a common 2.048 MHz external clock. A central STM32H7 microcontroller performs clock-driven acquisition, time-multiplexed LED sequencing via TLC5940 constant-current drivers, packet framing, and local SD card storage. For fNIRS operation, the currently active LED identifier is written into the ADS1299 status word for each sample, providing hardware-locked association between illumination state and acquired data. Framed data are forwarded to an ESP32-S2 coprocessor for Wi-Fi streaming, while USB connectivity is galvanically isolated to reduce conducted noise, mitigate ground-loop interference, and provide user safety if operation while plugged in is ever required. Power is supplied by a single-cell Li-ion battery with integrated charging and fuel-gauge monitoring.

## 2. System Architecture

This section describes the top-level architecture of the proposed EEG–fNIRS instrument, with emphasis on how clock-synchronous sampling and tight inter-modal synchronisation are achieved in hardware. The following subsections detail the timing guarantees, clocking strategy, and key design constraints that motivated the partitioning between acquisition and communication domains.

### 2.1. Timing and Synchronisation Guarantees

All time-critical operations in the proposed system are referenced to a single low-jitter master clock. A fixed-frequency CMOS oscillator (SG-8002 series) provides a 2.048 MHz clock that is distributed to all ADS1299 devices as their external conversion clock ($f_{\mathrm{CLK}}$). Driving all ADCs from a shared external clock enforces phase alignment across all 32 channels and eliminates inter-device drift that would otherwise arise from independent internal oscillators.

Acquisition timing on the STM32H7 is referenced exclusively to the ADS1299 data-ready (/DRDY) signal. Each /DRDY assertion triggers a fixed-latency, DMA-based SPI readout sequence, ensuring that all digitised signals (EEG electrodes, optical detector outputs, and auxiliary inputs) correspond to the same conversion instant. Network transport, coprocessor scheduling, and receiver-side timestamping therefore have no influence on the sampling timeline.

The fNIRS illumination sequence is derived from this same clock domain. LED pattern updates are advanced only at defined ADC sample boundaries, and illumination state transitions are phase-locked to integer multiples of the ADC sampling period. Short blanking intervals can be inserted between wavelength changes to reduce electrical and optical cross-coupling. For each conversion frame, the currently active illumination state (source index, wavelength, and blanking if applicable) is encoded directly into the per-sample metadata by overwriting the ADS1299 status word. This provides a hardware-level association between optical measurements and illumination state.

#### Inter-Modal Synchronisation and Effective fNIRS Rate

The system does not employ separate sampling clocks or independent ADCs for EEG and fNIRS. Instead, both EEG electrode voltages and optical detector voltages are digitised by the same ADS1299 ensemble on every conversion instant, locked to the shared master clock. The effective lower sampling rate of fNIRS signals arises solely from time-division multiplexing of the illumination sources and subsequent demultiplexing during analysis, rather than from a distinct low-frequency sampling process.

If the ADS1299 output data rate is $f_{s}$ and the illumination cycle contains $N_{\mathrm{state}}$ discrete LED states (including blanking intervals if used), the effective sample rate per state is

(1) $$f_{\mathrm{state}}=\frac{f_{s}}{N_{\mathrm{state}}}.$$

Wavelength- and source-specific optical time series are reconstructed by grouping samples according to their embedded state identifiers and may be decimated to conventional haemodynamic rates during analysis without introducing inter-clock drift. As a result, EEG and fNIRS data share a single hardware-defined sampling timeline, enabling direct cross-modal analysis without drift between independent sampling clocks at the acquisition source.

Software-level timestamping using the Lab Streaming Layer (LSL) is applied only for data transport and inter-device alignment at the receiver [7,8]. The key timing guarantee provided by the proposed hardware is a single clock-defined sampling timeline shared by EEG and optical channels. Quantitative characterisation of receiver-side timestamp uncertainty (including transport-dependent jitter and long-term drift during wireless streaming) remains the subject of ongoing work and will be reported in future studies.

### 2.2. Architectural Overview and Design Constraints

The instrument supports up to 32 simultaneously sampled analogue channels spanning EEG, fNIRS detector signals, and auxiliary biosignals. The architecture is divided into two functional domains:

1. A time-critical acquisition domain implemented on an STM32H7 microcontroller, responsible for all time-critical operations, including analogue sampling, LED sequencing, and local storage. 2. A communication domain implemented on an ESP32-S2, responsible for wireless networking, command handling, and data forwarding [9].

This partitioning is designed such that network latency, retransmissions, or scheduling on the wireless processor do not perturb sampling timing. All acquisition timing is referenced to a single master clock domain shared by the analogue front end and the acquisition controller.

The split also supports battery-powered operation: wireless radios dominate instantaneous power draw during streaming, so isolating Wi-Fi functionality on the ESP32-S2 allows it to be duty-cycled or disabled entirely (e.g., during local SD logging) without affecting the clock-driven acquisition loop on the STM32H7 [9,10].

From the outset, the design was constrained by three primary requirements: (i) low-jitter, clock-referenced sampling suitable for EEG; (ii) synchronised time-multiplexed fNIRS illumination and detection; and (iii) battery-powered operation with minimal analogue interference from digital subsystems.

### 2.3. Signal Acquisition and Timing Infrastructure

Electrophysiological and optical detector signals are digitised using Texas Instruments ADS1299 biopotential acquisition ICs [11]. Each ADS1299 provides eight simultaneous-sampling, 24-bit delta-sigma ADC channels with integrated programmable-gain amplifiers. Four devices are used to scale the system to 32 channels.

All ADS1299 devices are driven from a shared low-jitter external clock and operate with a common data-ready signal. This configuration guarantees phase alignment across all channels and across IC boundaries. Independent chip-select lines allow per-device configuration while preserving synchronous sampling behaviour.

Crucially, optical detector outputs from the fNIRS subsystem are routed into the same ADC infrastructure as EEG signals. By digitising both modalities with the same ADCs and clock domain, inter-modal synchronisation is achieved in hardware rather than via post hoc timestamp alignment. This approach avoids drift and quantisation error associated with multi-clock systems.

### 2.4. Optical Subsystem Integration

The fNIRS subsystem employs continuous-wave illumination using dual-wavelength LEDs, nominally centred near 750/850 nm, paired with integrated photodiode and transimpedance amplifier receivers [12,13]. Illumination is time-multiplexed such that only one wavelength is active at a given instant.

The illumination sequence is scheduled by the acquisition controller and is phase-locked to the ADC sampling clock. The current illumination state is embedded into the data stream, enabling unambiguous reconstruction of wavelength-specific optical signals during offline processing.

### 2.5. Processing, Storage, and Wireless Communication

The STM32H7 microcontroller performs all acquisition tasks, including SPI-based ADC readout using DMA, LED control sequencing, and packet framing. Acquired data are written concurrently to local SD card storage and streamed to the ESP32-S2 over a dedicated high-speed interface.

The ESP32-S2 runs FreeRTOS and handles Wi-Fi connectivity, command parsing, and network streaming. It does not participate in sampling or timing decisions. A simple flow-control protocol prevents buffer overrun and allows acquisition to be started and stopped under external control.

This separation increases system complexity but was chosen to preserve sampling-time stability under realistic wireless conditions and to ensure data integrity during mobile operation.

## 3. Hardware Implementation

This section summarises the hardware realisation of the platform, including the analogue front end, microcontroller and communication interfaces, and the PCB-level strategies used to support low-noise biopotential acquisition alongside time-multiplexed optical drive and high-speed digital I/O. The subsections highlight the main subsystems and implementation choices that most strongly impact signal integrity and practical deployability.

### 3.1. Analogue Front End

The electrophysiology analogue front end provides input protection, differential coupling, and low-noise regulation for biopotential acquisition. The ADS1299 programmable-gain amplifiers are configured to resolve microvolt-level EEG signals while maintaining sufficient dynamic range to accommodate auxiliary analogue inputs, including optical receiver channels. No fixed analogue RC anti-aliasing filter is employed at the inputs in order to preserve bandwidth flexibility across modalities. Instead, alias suppression and effective signal bandwidth limiting are provided by the ADS1299 delta-sigma architecture and its internal digital decimation filters, with the usable bandwidth set by the selected output data rate.

### 3.2. Microcontroller and Interfaces

The STM32H7 performs real-time acquisition using SPI and DMA, assembling sample frames from four ADS1299 devices. The ESP32-S2 provides Wi-Fi connectivity and serves as a network bridge between the instrument and external software. The STM32H7 was selected not only for its high-throughput SPI and DMA capabilities but also for its substantial computational headroom, enabling future deployment of on-device signal quality metrics, artefact detection, and lightweight edge machine learning models without altering the hardware-timed acquisition architecture.

The MCU selections were therefore driven by (i) processing requirements, (ii) peripheral availability, and (iii) power consumption. The STM32H7 provides sufficient processing headroom for real-time control and buffering, alongside the peripheral set needed for this design (multiple high-speed SPI interfaces with DMA support, timers for phase-locked sequencing, and an SD-card interface for local storage) [10]. The ESP32-S2 was chosen specifically to provide an integrated Wi-Fi radio and networking stack under FreeRTOS while keeping the acquisition timing domain independent [9]. In terms of power, this division permits low-power, fully untethered operation by allowing the wireless subsystem to be disabled during logging-only sessions and enabling streaming only when required; the resulting runtime differences between local logging and Wi-Fi streaming modes are reflected in the power characterisation results section.

### 3.3. PCB Layout and Isolation

**Figure 2:**
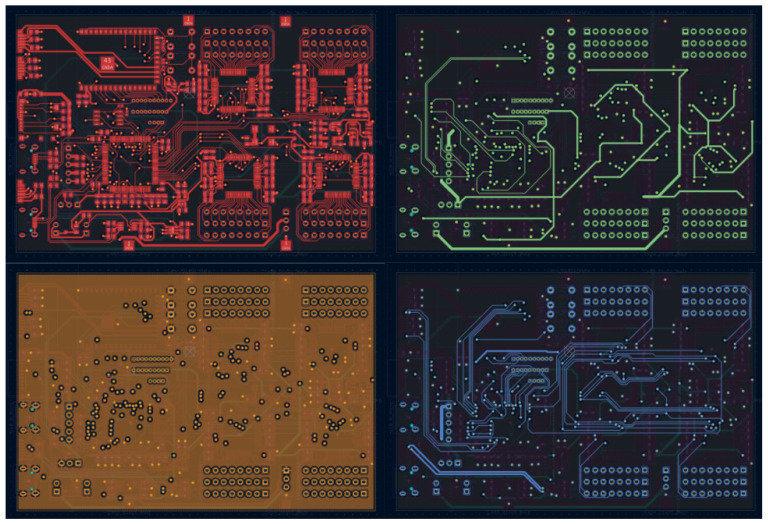
Layer-by-layer view of the four-layer printed circuit board for the proposed EEG–fNIRS instrument.

The system is implemented on a four-layer PCB with a signal, power, ground, and signal stack-up. Analogue, digital, and power regions are physically separated to reduce crosstalk. Galvanic isolation is implemented on the USB interface to reduce conducted noise and mitigate ground loops during tethered use. To support low-noise biopotential acquisition alongside high-speed digital communication and LED drive, the system is implemented on a four-layer PCB with strict domain separation (Figure 2). Layer 1 (top, red) of the proposed EEG–fNIRS instrument primarily routes low-level analogue signals associated with EEG electrodes, optical detector outputs, and ADS1299 front-end circuitry. Layer 2 (green) is used predominantly for power distribution and controlled routing of mixed-signal interconnects. Layer 3 (orange) forms a continuous ground plane, providing low-impedance return paths and shielding between analogue and digital domains. Layer 4 (bottom, blue) contains the majority of high-speed digital routing associated with the STM32H7 microcontroller, SPI buses, SD card interface, and ESP32-S2 communication subsystem. This stack-up, combined with physical separation of analogue, power, and digital regions, minimises crosstalk and conducted interference, supporting low-noise EEG acquisition alongside time-multiplexed optical drive and wireless communication.

**Figure 3:**
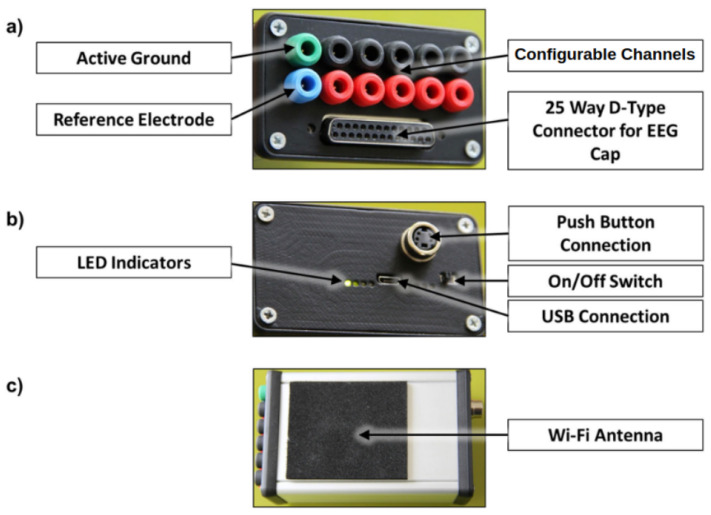
Hardware of the proposed EEG–fNIRS instrument: (**a**) front panel showing configurable analogue input channels, active ground and reference electrode connections, and a 25-way D-type connector for EEG cap interfacing; (**b**) rear panel showing LED status indicators, push-button connector, on/off power switch, and USB connection; (**c**) side view showing the external Wi-Fi antenna.

Figure 3 shows photographs of the developed smartphone-based EEG–fNIRS instrument system, illustrating (a) the top panel with D-Type connector for an EEG Cap, configurable channels that can be assigned as differential inputs (e.g., ECG, EOG) or used for fNIRS connections, (b) the bottom panel with LED indicators (from left to right: power, charging, connection status, and data activity), and (c) the side panel where the Wi-Fi Antenna is external to the case and protected with foam. The electronics and battery are housed in an aluminium enclosure.

### 3.4. Form Factor and Wearability

**Figure 4:**
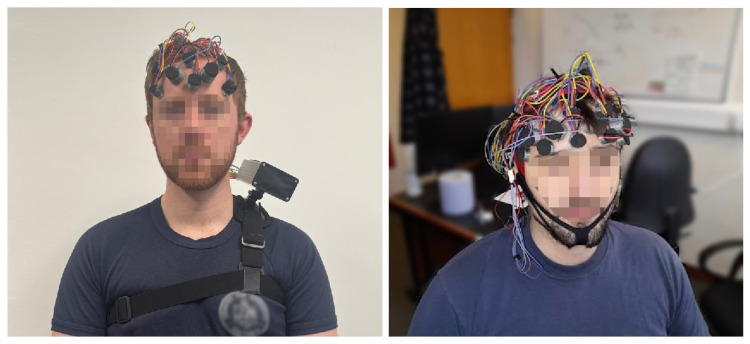
Wearable configurations of the proposed system. (**Left**): shoulder-mounted configuration during fNIRS-only operation. (**Right**): standard EEG cap configuration with fNIRS optodes positioned on the frontal scalp.

The complete device (electronics, battery, and aluminium enclosure) has outer dimensions of 120 mm × 78 mm × 43 mm and a total mass of 255 g, excluding electrodes, caps, and cabling (which are montage- and channel-count-dependent). The physical parameters of the developed device are summarised in Table 1. In typical use, the enclosure can be worn at the waist using the cable length of a conventional EEG head-cap with D-sub connector, or mounted on the shoulder using a standard action-camera quick-release mount (Figure 4).

In typical EEG operation, electrodes are placed using a standard 10–20 montage cap and connected via the D-sub interface shown in Figure 3. The ADS1299 bias drive is used in the conventional manner to set the subject common-mode voltage, and the reference electrode is selected according to the experimental montage (for example, mastoid or linked-mastoid reference). The 32 analogue inputs are software configurable and can be allocated as scalp EEG channels, auxiliary differential biosignals (e.g., ECG/EOG), or optical detector channels from the fNIRS subsystem.

Wearability was assessed qualitatively during short-duration recordings in office and laboratory environments, and the enclosure mass and connectorisation were selected to remain practical for waist- or shoulder-mounted use with standard cap cabling.

### 3.5. Optical Subsystem and LED Driver

The fNIRS illumination subsystem employs dual-wavelength near-infrared LEDs (L750/850-04A, Marubeni Corporation, Tokyo, Japan) [14] with nominal peak emissions at approximately 750/850 nm, wavelengths commonly used in continuous-wave fNIRS for separation of oxygenated and deoxygenated haemoglobin absorption. Each LED die is rated for a maximum forward current of 100 mA; however, in the present design, the drive current is limited by the TLC5940 constant-current sink drivers to a maximum of 60 mA per channel at a 3.3 V supply. Nominal operation is performed at 25–30 mA per wavelength to balance optical output power, thermal headroom, and battery life. At these operating currents, the LEDs provide a typical radiant output on the order of 20–30 mW per wavelength under continuous-wave operation.

LEDs are driven using TLC5940 devices (Texas Instruments Incorporated, Dallas, TX, USA) providing 16 channels of constant-current drive with 12-bit greyscale PWM control. Up to four TLC5940 devices are supported, enabling flexible optode layouts with up to 64 single-wavelength emitters or 32 dual-wavelength LED packages. Illumination is strictly time-multiplexed and phase-locked to the ADS1299 sampling clock, such that only one wavelength is active at any given instant. Short blanking intervals are inserted between wavelength transitions to reduce electrical and optical cross-coupling. The currently active LED (wavelength and source index) is embedded in the data stream, enabling unambiguous reconstruction of wavelength-specific optical signals during offline processing.

Optical detection is performed using integrated photodiode and transimpedance amplifier receivers (OPT101, Texas Instruments Incorporated, Dallas, TX, USA). The OPT101 integrates a silicon photodiode with a low-noise transimpedance amplifier in a single package and has been widely used in compact and wearable continuous-wave fNIRS systems due to its simplicity, stability, and low power consumption [15,16]. The photodetector exhibits a responsivity of approximately 0.3–0.6 A/W across the 750–850 nm spectral range, with peak responsivity near 850 nm. Based on the specified output noise of approximately 300 µV_RMS_ over a 0.1 Hz to 20 kHz bandwidth with a 1 MΩ transimpedance gain, this corresponds to an estimated noise-equivalent power (NEP) on the order of $10^{-12}$ W/$\sqrt{\mathrm{Hz}}$. This sensitivity is sufficient to resolve low-level diffuse reflectance signals following tissue attenuation while maintaining adequate dynamic range for short source–detector separations in continuous-wave fNIRS measurements.

### 3.6. Key Component Selection and Engineering Rationale

This section details the principal components used in the instrument and motivates their selection from an engineering perspective, with emphasis on noise performance, timing guarantees, and suitability for mobile multimodal acquisition.

#### 3.6.1. ADS1299 Biopotential and Auxiliary Analogue Front End

The ADS1299 (Texas Instruments Incorporated, Dallas, TX, USA) was selected as the primary acquisition IC because it provides eight channels of simultaneous-sampling, 24-bit delta-sigma conversion optimised for biopotential measurements [16,17]. The device integrates programmable-gain amplifiers, bias drive circuitry, and lead-off detection, substantially reducing external analogue complexity. Support for external clocking and a shared data-ready signal enables clock-synchronous operation across multiple devices, which is essential when scaling to 32 channels and when temporally aligning electrophysiological and optical measurements.

The ADS1299 offers low input-referred noise and a high common-mode rejection ratio (CMRR), specified at approximately −120 dB (typical) at 50/60 Hz, both of which are critical for scalp EEG acquisition in mobile and clinical environments. High CMRR ensures that voltages common to both differential inputs, such as 50/60 Hz mains interference and motion-induced artefacts, are strongly attenuated prior to amplification, preserving sensitivity to microvolt-level neural signals. The high input impedance minimises signal attenuation arising from electrode impedance mismatch, while flexible data-rate configuration enables application-specific trade-offs between signal bandwidth, noise performance, and power consumption.

#### 3.6.2. OPT101 Integrated Photodiode and Transimpedance Amplifier

The OPT101 integrates a photodiode and transimpedance amplifier in a single package, providing a voltage output proportional to incident optical power [18,19]. This integration simplifies the optical receiver design and improves channel-to-channel repeatability by avoiding discrete TIA component tolerances. The device offers sufficient bandwidth to support time-multiplexed illumination and enables observation of slow haemodynamic changes as well as higher-frequency optical pulsatility if desired.

The voltage output of the OPT101 can be routed directly into the ADS1299 input path, allowing optical intensity signals to be digitised using the same ADC infrastructure and timing as EEG channels.

#### 3.6.3. TLC5940 Constant-Current LED Driver

The TLC5940 (Texas Instruments Incorporated, Dallas, TX, USA) provides 16 constant-current sink channels with 12-bit greyscale PWM control and per-channel dot correction [20,21]. These features enable stable, repeatable LED current set points and compensation for emitter-to-emitter variation. The serial interface supports high update rates, allowing LED patterns to be refreshed at fixed points within the acquisition cycle. Integrated diagnostic features (open LED detection and thermal error flags) support fault detection during development and testing.

#### 3.6.4. Dual-Wavelength LED Emitters (750/850 nm)

Dual-wavelength LED emitters centred near 750/850 nm were selected to support conventional continuous-wave fNIRS measurements. These wavelengths lie within the optical window of biological tissue and provide differential sensitivity to oxygenated and deoxygenated haemoglobin [22]. The available radiant output at moderate drive currents supports an adequate signal-to-noise ratio while remaining compatible with a battery-powered thermal budget.

#### 3.6.5. Low-Jitter Master Clock Oscillator

A fixed-frequency low-jitter CMOS oscillator (SG-8002 series, Seiko Epson Corporation, Suwa, Nagano, Japan) is used as the master clock reference for the ADS1299. All ADC devices share this clock, ensuring phase alignment across channels and ICs. The LED sequencing schedule is derived from the same clock (or an integer multiple thereof), preventing long-term drift between electrophysiological sampling and optical multiplexing. This shared timing reference is a key design choice for preserving inter-modal synchronisation during extended recordings.

#### 3.6.6. USB Isolation and Power Subsystem

USB galvanic isolation (ADuM4160, Analog Devices, Inc., Wilmington, MA, USA) is incorporated to reduce conducted noise from host systems and to mitigate ground loops during tethered operation [23]. Portable operation is supported by a single-cell lithium-polymer battery and a dedicated linear charge controller (MCP73831/2 family, Microchip Technology Incorporated, Chandler, AZ, USA) which implements a CC/CV charge profile with minimal external components. A battery monitor (STC3100, STMicroelectronics, Geneva, Switzerland) with coulomb counting capability enables estimation of state-of-charge and supports logging of power state alongside physiological data.

### 3.7. Key Datasheet Specifications of Primary Components

Table 2 summarises the key datasheet specifications of the primary components used in the proposed EEG/fNIRS instrument, highlighting their roles in the system and the parameters most relevant to noise performance, timing, and power consumption.

### 3.8. Reference Analogue Front-End Performance (Datasheet)

This subsection provides datasheet-extracted reference values for the ADS1299 analogue front end to contextualise component selection and expected performance under ideal conditions.

For mobile EEG operation, rejection of common-mode interference is a primary design requirement due to the susceptibility of scalp electrodes to capacitive coupling from ambient power-line fields. The analogue front end is therefore designed around the ADS1299, which provides a theoretical common-mode rejection ratio (CMRR) of approximately −120 dB at 50/60 Hz under typical conditions (−110 dB minimum, datasheet). This level of CMRR is critical for suppressing mains interference arising from electrode impedance imbalance, body motion, and proximity to electrical infrastructure, thereby preserving sensitivity to microvolt-level neural signals without requiring aggressive analogue filtering or shielding.

The values in Table 3 are extracted from the ADS1299 datasheet and are provided as reference performance characteristics of the selected analogue front-end device. They represent expected limits under ideal conditions and are included to motivate the component selection and system architecture. End-to-end system noise, interference rejection, and channel isolation are additionally influenced by PCB layout, power distribution, shielding, and electrode interfaces. Comprehensive analogue benchmarking is outside the scope of the present work and will be addressed in future system characterisation.

## 4. Firmware and Data Protocol

This section describes the embedded firmware and data framing used to implement hardware-timed acquisition, device configuration, local logging, and network streaming. The subsections outline the STM32H7 acquisition pipeline, the division of responsibilities between the STM32H7 and ESP32-S2, and the receiver-side parsing and publication of the resulting data stream.

### 4.1. STM32H7 Deterministic Acquisition

The STM32H7 firmware implements a deterministic, clock-driven acquisition engine responsible for all time-critical operations, including synchronised ADC readout, LED sequencing, packet assembly, and local storage. Firmware execution is event-driven and centred on the ADS1299 data-ready (/DRDY) signal, which serves as the sole timing reference during active acquisition. Once streaming is initiated, the acquisition pipeline operates continuously and does not depend on higher-level sequencing or network state.

Device configuration, including channel enablement, programmable gain settings, sampling rate selection, and fNIRS illumination parameters, is performed prior to the start of streaming via a compact command packet transmitted from the ESP32-S2. These parameters are applied atomically before acquisition begins and remain fixed throughout a capture session, ensuring that all recorded data are internally consistent and directly comparable.

Time-multiplexed fNIRS illumination is implemented by updating the TLC5940 at defined points within the acquisition cycle, with short blanking intervals inserted between wavelength transitions. Each ADS1299 conversion frame consists of a 24-bit status word followed by 24-bit samples for each enabled channel. Upon receipt of a frame, the STM32H7 overwrites the status word with an encoded identifier corresponding to the currently active fNIRS LED (wavelength and source index). This operation binds the illumination state to the exact ADC sample in hardware time, enabling unambiguous demultiplexing of optical data at the receiver and eliminating reliance on software timestamps or packet ordering.

#### Hardware Settings, Signal Quality, and Preprocessing

During acquisition, each ADS1299 channel is configured in normal electrode-input mode with per-channel programmable PGA gain set via the CHnSET registers (firmware-configurable), and the ADS1299 internal reference buffer and bias drive are enabled (CONFIG3). The embedded firmware streams and logs raw 24-bit conversion frames without additional on-device digital preprocessing beyond the ADS1299 internal delta-sigma decimation filtering, and saturation is defined by the ADC reaching near full-scale codes (0x7FFFFF/0x800000), corresponding approximately to an input of $\pm (V_{\mathrm{ref}}/\mathrm{gain})$ at the ADS1299 inputs (device-dependent). For fNIRS illumination, LEDs are driven by TLC5940 constant-current sinks with 12-bit greyscale control (fixed-current operation, no automatic gain or auto-exposure). For optical channels, saturation corresponds to the OPT101 output (or the ADS1299 channel digitising it) approaching rail limits due to excessive optical power or ambient light. photo

### 4.2. STM32H7 Local Storage

Local logging is performed on the STM32H7 via an SD card interface concurrently with streaming and does not alter acquisition timing. The firmware writes framed packets that are identical (or trivially convertible) to those transmitted over Wi-Fi, simplifying offline analysis and preserving a complete acquisition record.

### 4.3. ESP32-S2 Wireless Streaming

The ESP32-S2 runs FreeRTOS and acts as a communication coprocessor. It configures an SPI interface as a high-throughput link to the STM32 and concurrently manages Wi-Fi and socket-level streaming. Command handling is implemented using a lightweight ASCII control protocol for actions, such as device setup, start streaming, and stop streaming.

During streaming, the ESP32 continuously receives framed packets from the STM32 and forwards them to an external client using a network socket. The ESP32-S2 does not participate in sampling decisions, timing control, or LED sequencing, and cannot influence acquisition timing once streaming has started. Error handling detects transmission failures and halts streaming to avoid uncontrolled buffer growth.

### 4.4. Receiver and LSL Publication

A lightweight companion application running on a PC or mobile device connects to the instrument over Wi-Fi (TCP to avoid packet loss), receives the streamed packets, parses the raw byte frames into channel-ordered samples, and publishes the data via LSL. The LSL stream is annotated with metadata describing channel count, sampling rate, and modality labels. Any LSL-compatible toolchain can then record (for example, via LabRecorder) or process the data in real time.

## 5. System Characterisation and Validation

This section reports functional characterisation and validation experiments that verify synchronised acquisition, timing integrity, and end-to-end compatibility with standard neurophysiology workflows. Electrical tests are first used to confirm multi-ADC synchronisation, followed by EEG and fNIRS demonstrations that establish physiologically plausible signals and analysis pipeline readiness.

### 5.1. Electrical Characterisation

**Figure 5:**
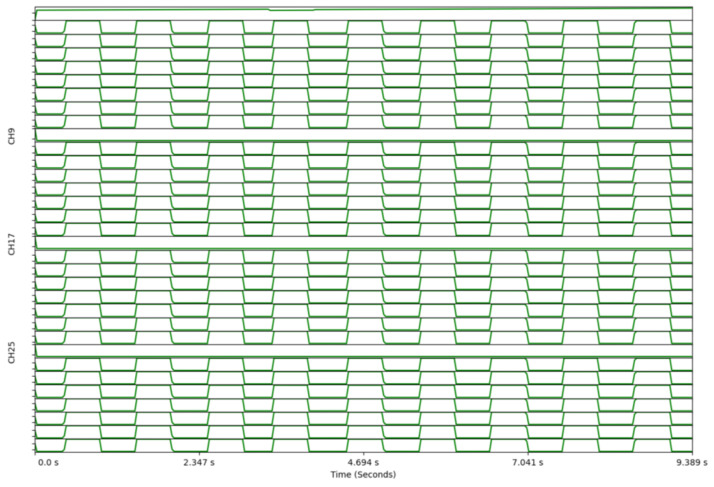
32-channel verification using the ADS1299 internal test signal. Internal square-wave test signal enabled on all four ADS1299 devices simultaneously, demonstrating consistent acquisition across 32 channels and validating synchronous sampling and readout using the shared external clock.

Electrical characterisation focused on the functional verification of synchronised acquisition, timing integrity, and physiological plausibility rather than exhaustive analogue benchmarking. As an initial end-to-end verification of multi-ADC readout, the ADS1299 internal test signal is enabled across all four devices and recorded concurrently (Figure 5).

This electrical verification confirms synchronous multi-ADC readout under the shared external clock. In the present work, we focus on the hardware-timed acquisition architecture and its end-to-end functional validation; detailed quantitative characterisation of receiver-side timestamp accuracy (including transport-dependent jitter and long-term drift during wireless streaming) remains an area for future study.

#### 5.1.1. Power Consumption and Battery Runtime

The instrument is powered by a single-cell lithium-ion battery with a nominal capacity of 2500 mAh at 3.7 V (approximately 9.25 Wh). After accounting for regulator inefficiencies and conversion losses across the analogue and digital supply rails, the usable energy available to the system is estimated at approximately 7.4 Wh. The effective usable energy depends on battery cutoff voltage and regulator efficiency, and the 7.4 Wh value is used here as a practical estimate for runtime calculation.

Overall power consumption is dominated by the digital processing and wireless communication subsystem, while the EEG analogue front end scales weakly with channel count. Each ADS1299 device contributes only a small incremental power increase, allowing the system to scale from low-channel-count EEG to full 32-channel operation without a proportional increase in energy demand.

The fNIRS illumination subsystem employs strict time-multiplexing, such that only a single infrared LED is active at any given instant, even when multiple sources are distributed across the optode array. As a result, the average power contribution of the illumination subsystem depends solely on the selected LED drive current and duty cycle, rather than on the total number of optodes present. This design choice simplifies both thermal management and battery-life estimation and decouples optical channel count from energy consumption.

Table 4 summarises measured end-to-end system power across representative operating modes. System current was measured at the battery input using a digital multimeter (121 GW) configured for DC current measurement. For each operating mode, measurements were recorded for at least 10 min following stabilisation to steady-state operation, and average power was computed from the mean current and the measured supply voltage; values therefore reflect average steady-state end-to-end consumption rather than transient peak currents. To avoid implying spurious precision given measurement uncertainty and supply-voltage variation, total power values are reported rounded to the nearest 10 mW. For streaming modes, measurements were performed off-body with the device positioned approximately 2 m from the receiver PC. The corresponding battery runtimes are estimated from the measured power and the effective usable battery energy (7.4 Wh).

Although multiple fNIRS source LEDs may be distributed across the optode array, only a single LED is enabled at any instant due to time-multiplexed operation. Consequently, the LED contribution to average power consumption and battery life is equivalent to that of a single continuously operating LED at the selected drive current, independent of the total number of optodes deployed.

Component-level current draw of the STM32H7, ESP32-S2, and ADS1299 subsystems was not separated in these measurements; therefore, Table 4 reports end-to-end system power across representative operating modes, which is the primary determinant of battery runtime.

#### 5.1.2. Thermal Stability of Optical Acquisition During Long-Term Operation

No appreciable thermal drift attributable to the OPT101 detector front-end or the TLC5940 LED driver was expected to impact haemodynamic measurements during recordings exceeding 1 h, because (i) LED drive current was regulated by constant-current sinks and (ii) sources were time-division multiplexed across multiple emitters (e.g., a cycle comprising 10 emitters), reducing the average dissipation per emitter to approximately 8–9 mW at 50 mA peak drive. Any residual warm-up drift is therefore expected to be slow and largely confined to the initial minutes after power-on, and was mitigated by baseline normalisation and detrending in the fNIRS processing pipeline.

### 5.2. Functional Validation

#### 5.2.1. EEG Validation: Resting-State Alpha Modulation

**Figure 6:**
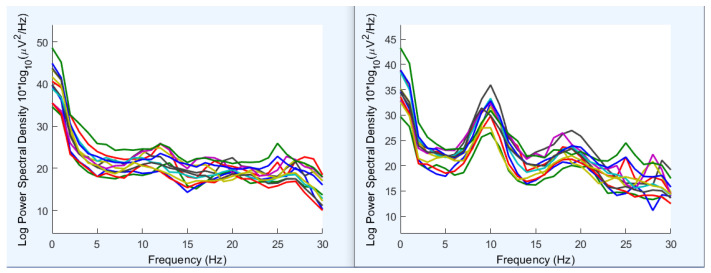
EEG validation: resting-state alpha modulation and EEGLAB compatibility. Power spectral density (PSD) estimates for eyes open (**left**) and eyes closed (**right**) conditions are shown, where each coloured trace represents an individual EEG channel. As expected, posterior alpha power is enhanced during eyes closed.

A resting-state protocol (eyes-open versus eyes-closed) was used to confirm expected modulation of occipital alpha power [24,25] and to demonstrate compatibility with standard EEG analysis workflows, including EEGLAB [26] (Figure 6). A single participant was recorded for five minutes with alternating 30-s eyes-open and eyes-closed blocks while fixating on a screen cross. Power spectral density (PSD) estimates were computed as a grand block average across the montage (O1, O2, T3, T5, Pz, Cz, C4, C3, P4, P3, T4, and T6). As expected, eyes-closed segments exhibited increased posterior alpha power (approximately 9–11 Hz), most prominently in occipital and parietal channels. PSDs were visualised in EEGLAB to confirm analysis pipeline compatibility.

#### 5.2.2. fNIRS Validation: Task-Evoked Haemodynamics

**Figure 7:**
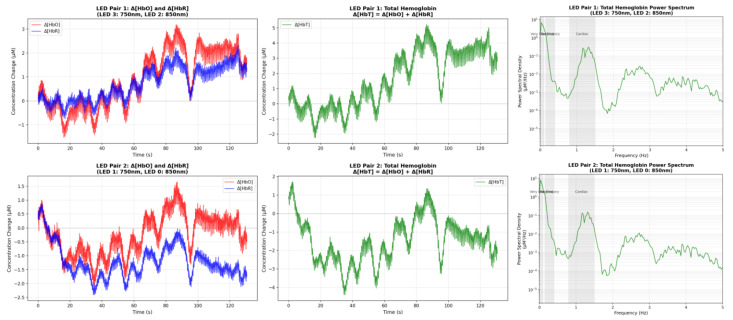
fNIRS validation: breath-hold haemodynamics and physiological spectral content. Time courses of ΔHbO/ΔHbR and ΔHbT (left/middle) and the ΔHbT power spectrum (right) during a breath-hold paradigm.

A breath-hold paradigm was used to validate detection of task-evoked changes in oxygenated and deoxygenated haemoglobin (HbO/HbR) and to confirm physiological spectral content indicative of good optical coupling [27,28] (Figure 7). Forehead fNIRS data were recorded during paced breathing followed by a deep inhalation at approximately 90 s. Concentration changes (ΔHbO, ΔHbR) were estimated for the 750/850 nm wavelength channels, and total haemoglobin was computed as ΔHbT = ΔHbO + ΔHbR. The ΔHbT power spectrum showed clear cardiac-band energy (approximately 1 Hz) and respiratory-band energy (approximately 0.2–0.4 Hz), consistent with strong optical coupling and physiologically plausible haemodynamic responses. While breath-hold responses demonstrate physiological sensitivity and effective optical coupling, future work will include task-evoked cortical activation paradigms.

#### 5.2.3. Combined Demonstration: ECG and Optical Pulsatility in LSL Viewer

**Figure 8:**
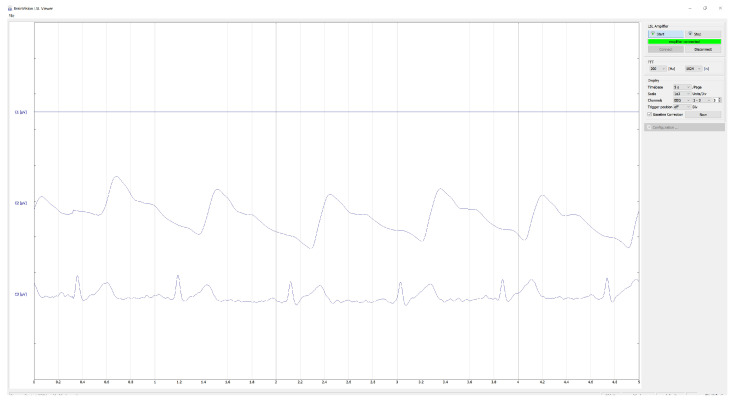
Real-time ECG and optical pulse (PPG) streaming via LSL. Screenshot of BrainVision LSL Viewer showing concurrent streaming of ECG and peripheral optical pulsatility.

As a system-level demonstration of synchronised multimodal streaming, an ECG signal digitised via the ADS1299 and a pulsatile optical signal acquired using the optical subsystem configured as a finger photoplethysmography (PPG) sensor were visualised concurrently in an LSL-compatible viewer [29,30] (Figure 8). Simple real-time filtering was applied in the viewer for visualisation. A consistent temporal offset is visible between the ECG R-peak and the subsequent optical pulse peak, as expected due to cardiovascular pulse transit time.

## 6. Comparative Analysis with Existing Mobile EEG–fNIRS Systems

Table 5 presents a comprehensive comparison of the proposed platform against representative commercial devices and research prototypes. Unlike commercial systems with fixed channel allocations (e.g., hard-wired EEG vs. fNIRS inputs), the proposed architecture features a “universal input” topology, allowing the 32 ADC channels to be dynamically reconfigured as EEG electrodes, fNIRS photodiodes, or a hybrid mix, supported by high-density optical stimulation drivers. As discussed earlier, fully integrated commercial EEG–fNIRS systems remain rare, and existing offerings are typically asymmetric combinations of a conventional EEG amplifier with a low-channel-count optical module; the comparison therefore includes both commercial systems and research-grade hybrids to provide a realistic view of current capabilities.

## 7. Discussion

This work is primarily an instrumentation contribution, and the validation results are intended to demonstrate (i) functional correctness of the timing and acquisition architecture, (ii) physiological plausibility of recorded EEG and optical signals, and (iii) end-to-end compatibility with established software workflows. Rather than optimising for maximal channel density, the design emphasises low-jitter, clock-synchronous sampling, hardware-enforced synchronisation between modalities, and a transparent data interface suitable for reproducible mobile experiments.

### 7.1. Interpretation of Validation Results

The 32-channel internal test-signal acquisition (Figure 5) verifies that the four ADS1299 devices operate as a synchronised acquisition ensemble under a shared external clock. In practical terms, this confirms consistent frame alignment across all channels and demonstrates that the SPI readout, DMA transfer, and packetisation pipeline preserve simultaneity at the scale required for multichannel EEG. This is a necessary prerequisite for multimodal analyses that depend on phase relationships or cross-channel temporal features.

The resting-state EEG demonstration (Figure 6) shows the expected increase in posterior alpha-band power during eyes-closed relative to eyes-open conditions, consistent with canonical observations in scalp EEG [24,25]. This result indicates that the system provides sufficient signal fidelity to recover a well-established neurophysiological marker using standard analysis tooling (EEGLAB) [26]. Importantly, this validation is intended as an end-to-end instrumentation check: the emergence of a stable alpha-band feature in the 9–11 Hz range provides evidence that baseline noise and interference were sufficiently low to recover microvolt-scale rhythms in a mobile-capable acquisition chain using standard tooling.

For the optical subsystem, the breath-hold experiment (Figure 7) demonstrates physiologically plausible haemodynamic behaviour and provides evidence of adequate optode coupling. Breath-hold paradigms are widely used to elicit robust cerebrovascular responses and to verify sensitivity to HbO/HbR dynamics in continuous-wave fNIRS [27,28]. The presence of cardiac- and respiration-band components in the optical spectrum further supports that the receiver chain captures meaningful physiological variability rather than being dominated by quantisation noise or drift. As with most breath-hold validations, the observed dynamics likely reflect a mixture of systemic and superficial haemodynamics in addition to any cortical contribution, and the experiment is not intended to localise task-evoked activation.

Finally, the concurrent ECG and optical pulsatility demonstration (Figure 8) provides a system-level check that multi-signal streaming is temporally coherent at the receiver and that the combined acquisition, transport, and LSL publication pipeline supports real-time multimodal visualisation. In this configuration, the optical channel is operated as a finger photoplethysmography (PPG) measurement rather than a conventional source-detector fNIRS channel. The observable delay between ECG R-peaks and the subsequent optical pulse is consistent with pulse transit time effects commonly reported in the PPG literature [29,30], providing an additional plausibility check on timing integrity across channels.

### 7.2. Comparison with Prior Mobile and Hybrid Systems

A recurring limitation in mobile hybrid EEG–fNIRS systems is the use of loosely coupled timing domains or post hoc alignment, which can introduce drift and ambiguity when combining modalities [3,12]. Compared to the earlier smartphone-based EEG platform [6], the present work adds synchronised optical acquisition within the same sampling clock domain and increases on-device processing headroom (STM32H7) while keeping wireless communication isolated on a dedicated Wi-Fi coprocessor (ESP32-S2).

Architecturally, the present system aligns most closely with designs that enforce synchronisation through a shared sampling clock and unified acquisition pipeline, such as the M3BA architecture reported by von Lühmann et al. [16]. The present work extends this approach to higher EEG channel counts (up to 32) while also embedding illumination state directly into the acquired data stream, which reduces reliance on software timestamps for optical demultiplexing and supports unambiguous reconstruction during offline analysis. Compared to compact colocated EEG/fNIRS sensors that focus on minimal channel counts and specific optode geometries [36], the proposed platform prioritises configurability and explicit interface definition, enabling a broader range of experimental setups at the cost of increased system complexity.

### 7.3. Limitations and Future Characterisation

The current validation consists of single-participant demonstrations focused on functional verification and physiological plausibility rather than exhaustive analogue benchmarking. In particular, we did not perform standardised input-referred noise measurements with shorted inputs, systematic motion-sensitivity studies, or controlled comparisons of 50/60 Hz susceptibility across environments. In addition, although event-related potentials such as visual evoked potentials (VEPs) are a common benchmark for EEG timing and amplitude fidelity, VEP experiments were not included in the present evaluation and will be addressed in future work alongside multi-subject studies.

Future characterisation will therefore include (i) standardised noise and interference measurements under controlled electrode impedance conditions, (ii) quantitative timing analysis of inter-modal skew and jitter under streaming and logging modes, and (iii) task-evoked EEG and fNIRS paradigms with established expected responses, including VEP/ERP protocols and functional activation studies. To fully leverage the portability of the platform, future validation will also target ambulatory and naturalistic protocols (for example, walking, dual-task paradigms, and field deployments) to quantify robustness under motion and variable electromagnetic conditions.

Finally, the computational headroom of the STM32H7 provides a pathway to implement on-device signal-quality metrics, artefact detection, and lightweight edge machine learning models for real-time assessment and adaptive acquisition (for example, automated channel-quality scoring, motion/artefact flagging, or closed-loop adjustment of illumination and gain), while preserving the clock-driven acquisition architecture described in this work.

## 8. Conclusions

This work presents a compact EEG/fNIRS instrument that supports clock-synchronous acquisition of up to 32 channels, with concurrent local SD storage and Wi-Fi streaming exposed to external software via the Lab Streaming Layer (LSL). The primary contribution is an instrumentation architecture that enforces inter-modal synchrony in hardware using a shared master clock and unified ADC sampling timeline, while keeping wireless transport on a separate coprocessor so that network behaviour cannot perturb sampling timing. For fNIRS, embedding illumination state directly into the sampled data stream provides an unambiguous association between optical measurements and LED sequencing during both streaming and offline analysis.

Functional validation demonstrates end-to-end operation and physiological plausibility: synchronised 32-channel acquisition using the ADS1299 internal test signal, recovery of canonical resting-state alpha modulation using standard EEG analysis tooling, breath-hold fNIRS responses with expected haemodynamic dynamics and physiological spectral content, and concurrent ECG/optical pulsatility streaming and visualisation via LSL. While these demonstrations are not intended as exhaustive analogue benchmarking, they establish a practical, reproducible platform for mobile multimodal experiments and provide a clear basis for systematic future characterisation.

Future work will quantify noise, interference rejection, and inter-modal timing performance under controlled conditions and during ambulatory use, and will extend validation to established task-evoked EEG and fNIRS paradigms across multiple participants. The available computational headroom on the STM32H7 further enables on-device signal-quality metrics and lightweight edge processing while preserving the clock-driven acquisition design.

## Figures and Tables

**Table 1:** Physical parameters of the developed device.

Parameter	Value
Mass (device only; battery + enclosure)	255 g
Dimensions (L × W × H)	120 mm × 78 mm × 43 mm

**Table 2:** Key datasheet specifications of primary components used in the proposed EEG/fNIRS instrument.

Component	Role in System	Key Parameters (Datasheet)
TI ADS1299	EEG and auxiliary analogue acquisition	8 differential channels per IC; 24-bit resolution; data rates 250–16,000 SPS ($f_{\mathrm{CLK}}=2.048$ MHz); PGA gains 1–24; input-referred noise ≈0.57 μV_RMS_ (250 SPS, gain 24), ≈1 μV_pp_ (0.01–70 Hz, gain 24); CMRR −110 dB min (50/60 Hz); input impedance ≈1 GΩ; external clock 1.5–2.25 MHz; power ≈40 mW typ (bias off)
TI OPT101	Optical receiver (photodiode + TIA)	Spectral responsivity ≈0.45 A/W at 650 nm (reduced at 750–850 nm); output responsivity ≈0.45 V/μW (1 MΩ internal); bandwidth ≈14 kHz; supply 2.7–36 V; quiescent current ≈120 μA; output noise ≈300 μV_RMS_
TI TLC5940	Constant-current LED driver	16 channels; 12-bit greyscale PWM; 6-bit dot correction; constant-current range up to 120 mA; LED supply up to 17 V; serial interface up to 30 MHz; open-LED and thermal diagnostics
Dual-wavelength LED (750/850 nm)	fNIRS illumination	Peak wavelengths 750/850 nm; radiant power ≈8–18 mW at 50 mA; forward voltage ≈1.55–1.85 V typ; max forward current 100 mA; common-anode package
SG-8002 oscillator	Master sampling clock	CMOS output; frequency range 1–125 MHz; supply 3.0–5.0 V; frequency tolerance ±50 to ±100 ppm; jitter ≤250 ps p–p; startup ≤10 ms
ADuM4160	USB galvanic isolation	Isolation rating 5000 V_RMS_ (1 min); USB low/full speed (1.5/12 Mbps); CMTI >25 kV/μs; IEC/EN 60601-1 compliant
MCP73831/2	Li-ion charge control	CC/CV algorithm; regulation voltage options 4.2–4.5 V (±0.75%); programmable charge current 15–500 mA; thermal regulation and shutdown
LP785060 LiPo cell	Portable power	Nominal 3.7 V; capacity 2500 mAh typ; max continuous discharge 1.5 A; charge cutoff 4.2 V; discharge cutoff 3.0 V
STC3100	Battery monitoring/coulomb counting	Battery voltage, temperature, and current monitoring; ±80 mV sense range; coulomb counter resolution ≈0.2 mAh; I^2^C interface; operating current ≈100 µA typ

**Table 3:** Datasheet-extracted analogue performance of the ADS1299 (reference values). PSRR: power-supply rejection ratio; CMRR: common-mode rejection ratio.

Parameter	Datasheet Value	Practical Implication for This System
CMRR (50/60 Hz)	−120 dB typ (−110 dB min)	Strong rejection of mains interference under electrode impedance imbalance
Input-referred noise (250 SPS, gain 24)	$0.57\;\mu V_{\mathrm{RMS}}$	Below typical scalp EEG amplitudes
Input-referred noise (500 SPS, gain 24)	$0.81\;\mu V_{\mathrm{RMS}}$	Supports higher-bandwidth EEG
DC input impedance	1000 MΩ	Minimises electrode loading
Input bias current	±300 pA	Limits electrode polarisation drift
Dynamic range	≈129 dB	Supports microvolt EEG and auxiliary signals
PSRR (50/60 Hz)	96 dB	Reduces supply-coupled interference
Channel crosstalk	−110 dB	Preserves channel independence

**Table 4:** Measured end-to-end power consumption and estimated battery runtime across operating modes. LED subsystem is time-multiplexed; one LED active at any time.

Operating Mode	MCU	ADS1299 Count	IR LED State	Total Power (mW)	Estimated Runtime (h)
EEG-only, local storage	STM32	1	None	280	26.4
EEG-only, local storage	STM32	4	None	400	18.5
EEG + fNIRS (nominal)	STM32	4	1 LED @ 25–30 mA	470	15.7
EEG + fNIRS (high optical)	STM32	4	1 LED @ 50–60 mA	530	14.0
EEG-only streaming	STM32 + ESP32	1	None	590	12.5
EEG-only streaming	STM32 + ESP32	4	None	710	10.4
EEG + fNIRS streaming (nominal)	STM32 + ESP32	4	1 LED @ 25–30 mA	780	9.5
EEG + fNIRS streaming (high optical)	STM32 + ESP32	4	1 LED @ 50–60 mA	840	8.8

**Table 5:** Comparison of representative mobile/wearable EEG, fNIRS, and hybrid systems. Commercial specifications reflect manufacturer-reported maxima for portable configurations.

System	Config. (Channels)	Performance ($f_{s}$/Noise)	Connectivity & Sync	Physical (Bat. /Mass)
Commercial Systems (Fixed Architecture)				
Brain Products LiveAmp [31]	EEG only 8–64 ch (discrete models)	$f_{s}$: Up to 1 kHz ^†^ Noise: $<2\;\mu V_{\mathrm{pp}}$ (input-referred, vendor spec.)	RF: 2.4 GHz Proprietary Sync: Trigger In / LSL Store: Onboard SD	∼3.5–4.5 h Wearable Amp (60–120 g, model-dependent)
g.tec g.Nautilus [32]	EEG + fNIRS Fixed: 8–64 EEG + 8 fNIRS	$f_{s}$: EEG 500 Hz; fNIRS ∼10 Hz Noise: Not reported	RF: 2.4 GHz Proprietary Sync: Base Station (8-bit) Store: No standalone storage (PC/base station required)	∼1.5–8 h (config.-dependent) Integrated Cap (weight n/a)
NIRx NIRSport2 [33,34]	fNIRS only 16 Sources/16 Detectors (∼40–60 ch single dev.)	$f_{s}$: Max 240 Hz (config. dep.) Dyn. Range: >80 dB (meas.)	RF: Wi-Fi/USB Sync: LSL/TTL Input Store: Onboard	5–6 h Portable Unit ∼900–970 g (config.-dependent)
Research Prototypes				
Li et al., 2024 [35]	EEG + fNIRS Fixed: 2 EEG + 10 fNIRS	Not fully reported	RF: Wireless Sync: Claimed Sync.	Forehead Patch
von Lühmann (M3BA) [16]	EEG + fNIRS Fixed: 4 EEG + 2 fNIRS	$f_{s}$: EEG 500 Hz; fNIRS 16.7 Hz Noise: Not reported	RF: Bluetooth Sync: Shared ADC Clock Store: MicroSD	Modular Nodes
Proposed Platform				
This work	32 Universal Inputs (Software-selectable as all EEG, all fNIRS, or mix) + 64 LED Drivers (32 Dual-λ Sources)	$f_{s}$: EEG 16 kSPS / fNIRS Sync EEG Noise: 0.57 µV_RMS_ (datasheet) ^‡^ fNIRS NEP: $∼1\times 10^{-12}\;W/\sqrt{\mathrm{Hz}}$	RF: Wi-Fi (LSL / TCP) Sync: Shared ADC clock + embedded LED state; LSL (transport) Store: Onboard SD	8.8–12.5 h Streaming 14.0–26.4 h Logging 255 g; 120 × 78 × 43 mm

^†^ 1000 Hz supported on 32 channels; 64 channels often limited to 500 Hz. ^‡^ Datasheet-specified input-referred noise for ADS1299 (250 SPS, Gain 24).

## Data Availability

Data available upon reasonable request.

## References

[1] Pinti P., Aichelburg C., Gilbert S., Hamilton A., Hirsch J., Burgess P., Tachtsidis I. (2018). A Review on the Use of Wearable Functional Near-Infrared Spectroscopy in Naturalistic Environments. Jpn. Psychol. Res..

[2] Finnis R., Mehmood A., Holle H., Iqbal J. (2025). Exploring Imagined Movement for Brain–Computer Interface Control: An fNIRS and EEG Review. Brain Sci..

[3] Ahn S.-K., Jun S. (2017). Multi-Modal Integration of EEG–fNIRS for Brain–Computer Interfaces: Current Limitations and Future Directions. Front. Hum. Neurosci..

[4] Ghosh S., Máthé D., Harishita P., Sankarapillai P. (2023). Review of Multimodal Data Acquisition Approaches for Brain–Computer Interfaces. BioMed.

[5] Cui W., Lin K., Liu G., Sun Y., Cai J. (2024). A Wireless Integrated EEG–fNIRS System for Brain Function Monitoring. IEEE Sens. J..

[6] Bateson A.D., Asghar A.U.R. (2021). Development and Evaluation of a Smartphone-Based Electroencephalography (EEG) System. IEEE Access.

[7] Kothe C., Shirazi S.Y., Stenner T., Medine D., Boulay C., Grivich M.I., Artoni F., Mullen T., Delorme A., Makeig S. (2025). The Lab Streaming Layer for Synchronized Multimodal Recording. Imaging Neurosci..

[8] Kothe C., Shirazi S.Y., Stenner T., Medine D., Boulay C., Grivich M.I., Artoni F., Mullen T., Delorme A., Makeig S. Lab Streaming Layer (LSL) GitHub Repository. https://github.com/sccn/labstreaminglayer.

[9] Espressif Systems (2025). ESP32-S2 Series Datasheet: Wi-Fi SoC with USB and Security Features.

[10] STMicroelectronics (2025). STM32H743VI Datasheet: High-Performance ARM Cortex-M7 Microcontroller.

[11] Texas Instruments (2014). ADS1299: Low-Power, 8-Channel, 24-Bit Analog Front-End for Biopotential Measurements.

[12] Uchitel J., Vidal-Rosas E., Cooper R., Zhao H. (2020). Wearable, Integrated EEG–fNIRS Technologies: A Review. Sensors.

[13] Mohamed M., Jo E., Mohamed N., Kim M., Yun J., Kim J. (2020). Development of an Integrated EEG/fNIRS Brain Function Monitoring System. Sensors.

[14] Marubeni Component Solutions (2025). L750/850-04A Dual-Wavelength Near-Infrared LED Datasheet.

[15] Arivudaiyanambi J., Mohan S., Cherian S.M., Natesan K. (2020). Design of a Wearable Four-Channel Near-Infrared Spectroscopy System for the Measurement of Brain Hemodynamic Responses. Biomed. Eng. Biomed. Tech..

[16] von Lühmann A., Wabnitz H., Sander T., Müller K.-R. (2017). M3BA: A Mobile, Modular, Multimodal Biosignal Acquisition Architecture for Miniaturized EEG–NIRS-Based Hybrid BCI and Monitoring. IEEE Trans. Biomed. Eng..

[17] Lee S., Shin Y., Kumar A., Kim M. (2017). Dry Electrode-Based Fully Isolated EEG/fNIRS Hybrid Brain-Monitoring System. Proceedings of the IEEE Engineering in Medicine and Biology Society (EMBC).

[18] Li T., Zhong F., Pan B., Li Z., Huang C., Deng Z. (2016). A Brief Review of OPT101 Sensor Application in Near-Infrared Spectroscopy Instrumentation for Intensive Care Unit Clinics. Sensors.

[19] Texas Instruments (2025). OPT101 Datasheet: Photodiode with Integrated Transimpedance Amplifier.

[20] Gurel N.Z., Jung H., Hersek S., Inan O.T. (2019). Fusing Near-Infrared Spectroscopy with Wearable Hemodynamic Measurements Improves Classification of Mental Stress. IEEE Sens. J..

[21] Texas Instruments (2025). TLC5940: 16-Channel, 12-Bit PWM LED Driver with Dot Correction and Serial Interface.

[22] Tuchin V.V. (2015). Tissue Optics: Light Scattering Methods and Instruments for Medical Diagnostics.

[23] González-Briceño G., Medow J. (2017). Design and Evaluation of a USB Isolator for Medical Instrumentation. Proceedings of the IEEE Engineering in Medicine and Biology Society (EMBC).

[24] Barry R.J., Clarke A.R., Johnstone S.J., Magee C.A., Rushby J.A. (2007). EEG Differences Between Eyes-Closed and Eyes-Open Resting Conditions. Clin. Neurophysiol..

[25] Lopes da Silva F. (2011). EEG and MEG: Relevance to Neuroscience. Neuron.

[26] Delorme A., Makeig S. (2004). EEGLAB: An Open Source Toolbox for Analysis of Single-Trial EEG Dynamics Including Independent Component Analysis. J. Neurosci. Methods.

[27] Scholkmann F., Kleiser S., Metz A.J., Zimmermann R., Wolf U., Wolf M. (2014). A Review on Continuous-Wave Functional Near-Infrared Spectroscopy and Imaging Instrumentation and Methodology. NeuroImage.

[28] Tachtsidis I., Scholkmann F. (2016). False Positives and False Negatives in Functional Near-Infrared Spectroscopy: Issues, Challenges, and the Way Forward. Neurophotonics.

[29] Allen J. (2007). Photoplethysmography and Its Application in Clinical Physiological Measurement. Physiol. Meas..

[30] Zhang G., Hahn J.-O., Mukkamala R. (2011). Continuous and Noninvasive Estimation of Arterial Blood Pressure via Pulse Transit Time. Physiol. Meas..

[31] Brain Products GmbH (2025). LiveAmp: Mobile Wireless EEG Amplifier.

[32] g.tec Medical Engineering GmbH (2025). g.Nautilus fNIRS: Wireless EEG–fNIRS Hybrid System.

[33] NIRx Medical Technologies LLC (2025). NIRSport2: Portable Wearable fNIRS System.

[34] NIRx Medical Technologies LLC NIRSport 2: Portable, Whole-Head, High-Density fNIRS (Product Brochure, Rev. 18 October 2018). https://nirx.net/s/20181018-NIRSport2Brochure-web.pdf.

[35] Li B., Li M., Xia J., Jin H., Dong S., Luo J. (2024). Hybrid Integrated Wearable Patch for Brain EEG–fNIRS Monitoring. Sensors.

[36] Hameau F., Planat-Chrétien A., Gharbi S., Prada-Mejia R., Thomas S., Bonnet S., Rascle A. (2025). Compact Colocated Bimodal EEG/fNIRS Multi-Distance Sensor. Sensors.

